# Hot ambient conditions shift the Force / EMG relationship

**DOI:** 10.1186/2193-1801-2-317

**Published:** 2013-07-15

**Authors:** Sebastien Racinais

**Affiliations:** Aspetar, Qatar Orthopaedic and Sports Medicine Hospital, Research and Education Centre, PO Box 29222, Doha, Qatar

**Keywords:** Temperature, Hyperthermia, Muscle, Exercise, Heat

## Abstract

**Purpose:**

This manuscript reports the data from two interventions on human subjects aiming to determine the effect of elevated core (HOT-core, study 1, 14 participants) and skin (HOT-skin, study 2, 11 participants) temperature on the force / EMG relationship.

**Methods:**

In both studies, participant underwent an experimental trial and a control (CON) trial, in which maximal voluntary contractions (MVC) of the plantar flexors, surface EMG recordings of both the *soleus* and *gastrocnemius medialis* (GM), and electrical stimulation of the *tibial nerve* were performed to determine the percentage of voluntary activation (VA). During the HOT-core trial, rectal temperature was passively increased and then clamped at 39°C by adjusting the room temperature in the range of 46-50°C. During the HOT-skin trial, tests were performed in a temperate environment (~20°C) and skin temperature was locally affected by applying a cool or a hot pack during 1 min.

**Results:**

HOT-core resulted in a decrease in MVC torque (−19%) and VA (−5%) (p < 0.05). HOT-skin did not induce any changes in MVC torque (−1%) or VA (+0%). However, the EMG activity (RMS) was decreased both in HOT-core (*soleus −*40%, GM −33%) and HOT-skin (*soleus −*10%, GM −13%), compared with CON (p < 0.05).

**Conclusion:**

The effect of skin temperature on EMG recordings may be attributed to both methodological and physiological factors. Hot ambient conditions shift the Torque / EMG relationship to the left, with the decrement in torque induced with passive hyperthermia lower than the decrement in EMG.

## Introduction

Whilst some studies have observed a decrease in electromyographic (EMG) activity while cycling in hot ambient conditions (Kay et al. 2001; Tucker et al. 2004; Tucker et al. 2006), others have reported that EMG activity was not affected during running (Ftaiti et al. 2001) and cycling (Hunter et al. 2002). However, it is difficult to interpret such EMG data during whole-body dynamic exercise as modifications in EMG activity may be linked to alterations in exercise modality, pattern and intensity. For example, it has recently been reported that EMG activity was not affected by hot ambient conditions during the resting phase of the cycling stroke or during the pushing phase at a given intensity, but was lower at exhaustion in hot, compared with temperate conditions (Racinais and Girard 2012). Nevertheless, this study concluded that the changes in EMG where not triggered by a neurophysiological failure, but were a side effect of the lower power output due to the cardiovascular and perceptual strain during active hyperthermia. Therefore, the effect of temperature upon muscle electrical activity in isolation may be questioned, potentially limiting the interpretation of EMG data during whole-body dynamic exercise.

Some groups have performed neuromuscular testing based on maximal voluntary isometric contractions (MVC) pre- and post-whole-body exercise, reporting lower EMG activity during MVCs following exercise in hot environment compared to temperate, both during prolonged (Nybo and Nielsen 2001) and repeated contractions (Martin et al. 2005). However, this finding was not observed during brief MVCs (Nybo and Nielsen 2001; Saboisky et al. 2003; Racinais and Girard 2012; Girard et al. 2013). Exercise-induced fatigue might act as a confounding factor in these studies; and thus EMG activity should be analyzed during passive hyperthermia to isolate the effect of temperature on muscle electrical activity.

Passive hyperthermia has been shown to reduce maximal muscle force as well as voluntary activation, estimated by the twitch interpolation technique (Morrison et al. 2004; Thomas et al. 2006; Racinais et al. 2008; Périard et al. 2011). This reduction suggests that the electrical activity of the muscle during a MVC would also be reduced in hot ambient conditions. Unfortunately, these studies did not report the muscle electrical activity. Therefore, the aim of this study was to investigate the effect of passive hyperthermia on the EMG response during isolated isometric contractions. It is hypothesized that passive hyperthermia would decrease maximal force and voluntary activation, and that these decrements would be related to a reduction in muscle electrical activity. In addition, a second experiment was performed to verify if any potential changes in the EMG signals could be attributed to the temperature of the skin – electrode pair independently of hyperthermia.

## Methods

### Participants

Fourteen participants (7 males and 7 females, aged 32 ±2 yr, weight 67.8 ±10.6 kg and height 170 ±6 cm) participated in the study 1. Eleven participants (8 males and 3 females, age 31 ±4 yr, weight 71.4 ±11.9 kg and height 179 ±8 cm) participated in the Study 2. None of the participants suffered from injuries at the time of the experiment and they were required to avoid all vigorous activity for the 24 h preceding each trial. The project was approved by the Aspetar scientific committee and by the Shafallah ethics committee. The procedures complied with the Declaration of Helsinki regarding human experimentation. Written informed consent was obtained from all the participants before the beginning of the testing.

### General procedure

Participants completed neuromuscular assessments in both a control condition (CON) and a condition involving either generalized (study 1, HOT-core) or localized (study 2, HOT-skin) heating. Neurophysiological assessments included brief (~4-5 s) maximal (MVC, with strong verbal encouragement) and submaximal (from 25% to 75% of MVC) voluntary isometric contractions of the *plantar flexors* (PF) (see below for details).

Superimposed percutaneous electrical stimulations (400 V, rectangular pulse of 1 ms) were delivered during the MVCs by a high-voltage stimulator (Digitimer DS7AH, Digitimer, Hertfordshire, England) over the *tibial nerve*. The cathode electrode was located in the popliteal cavity (with a constant pressure supplied by a strap) and the anode distal to the patella. Placements of the electrode were marked with a permanent marker and kept constant throughout the experiment. The intensity of stimulation was determined for each participant at the beginning of the experiment by progressively increasing amperage (10 mA increments) until there was no further increases in either peak twitch mechanical response, or concomitant electrophysiological response (M-wave). This intensity was further increased by 50% (i.e., supra-maximal) to ensure that all stimulations were performed on the plateau of response and kept constant thereafter.

Voluntary activation (VA) during MVCs was estimated from the mechanical responses to a superimposed and a potentiated twitch as VA (%) = (1-Superimposed Twitch/Potentiated Twitch) × 100 (Merton 1954). All assessments were performed on the right leg, with the participants in a seated position with the ankle and the knee flexed at 90° and 100°, and the foot securely strapped to a fixed custom-made dynamometric pedal. The torque was recorded over a 1 s of plateau along with the concomitant EMG activity. Each test was repeated three to six times (see details below) and data were averaged for analyses.

### Testing procedure of study 1

Following a familiarization trial, participants underwent a CON (room set at 24°C and 35% Relative Humidity (RH)) and a HOT-core (room set at 50°C and 35% RH) trial separated by 4-7 days. The two experimental trials were performed in an environmental room (Tescor, Warminster, PA, USA), in a counter-balanced order, at the same time-of-day, with subjects wearing the same attire (i.e., shorts and t-shirt). Prior to each trial, participants rested for 1 h (laboratory at ~24°C) and drank 500 ml of water while they were equipped with rectal and skin temperature probes, and EMG surface electrodes (see below for details). Participants provided a urine sample. If the urine specific gravity (URC-NE, Atago, Tokyo, Japan) was above 1.020 g/ml the trial was postponed and the participants were requested to drink more water.

The trial began by a controlled similar activity (10 min of walking on a treadmill at 4 km.h^-1^) followed by ~45-60 min of rest in a seated position (corresponding to the time needed to reach a core temperature of 39°C in the HOT-core trial). Thereafter, the neurophysiological assessments included three sets of contraction (MVC with VA determination followed by submaximal contractions at 50%, 25% and 75% of MVC). For each submaximal contraction, the target torque was calculated from the preceding MVC and displayed on the screen. Doublet stimulations (20 Hz) were applied to determine VA during the MVCs and record the superimposed M-wave.

### Testing procedure of study 2

All tests were performed in temperate ambient conditions (laboratory ~20°C). The CON neurophysiological assessments were performed using cool EMG electrode (storage temperature ~5°C) applied on cool skin (1 min of a cool pack application, see Table 1 for values). The HOT-skin neurophysiological assessments were performed using warm EMG electrode (storage temperature ~34°C) applied on a warm skin (1 min of a warm pack application, see Table 1 for values). The two experimental trials were performed in a counter-balanced order, on the same day, with subjects wearing the same attire (i.e., shorts and t-shirt). Prior to commencement, participants were equipped with skin temperature probes and EMG surface electrodes (see below for details). Three MVC were performed. Single stimulations were applied to determine VA during the MVCs and record the superimposed M-wave.

**Table 1:** **Torque / EMG relationship responses to an increase in core and/or skin temperature**

	Study 1		Study 2	
	CON	HOT-core	CON	HOT-skin
*Temperature responses*				
Core temperature (°C)	37.3 ±0.4	39.0 ±0.2*	-	-
Skin temperature - Soleus (°C)	28.8 ±0.4	39.8 ±0.9*	25.4 ±2.2	35.2 ±0.9*
Skin temperature - GM (°C)	30.1 ±0.4	39.8 ±0.7*	23.8 ±2.0	35.6 ±1.3*
*Torque / RMS ratio (MVC)*				
Soleus (N.m.mV^-1^)	472 ±260	638 ±327*	1297 ±443	1416 ±473*
GM (N.m.mV^-1^)	245 ±135	299 ±168*	1405 ±931	1551 ±933*
*Slope Torque/RMS relationship*				
Soleus (N.m.mV^-1^)	576 ±283	768 ±204*	-	-
GM (N.m.mV^-1^)	291 ±146	345 ±111*	-	-

CON: normo thermic control condition. HOT-core: passive hyperthermia in hot ambient conditions. HOT-skin: local modification of the skin temperature. RMS: Root Mean Square of the electromyographic signal. * p < 0.05.

### Recording

#### Electromyography

For both studies, bipolar EMG signals were recorded over the muscle belly of the *Soleus* via Ag/AgCl electrodes (Ambu Blue sensor T, Ambu A/S, Denmark) with a diameter of 9 mm and a between electrode distance of 3 cm. In addition, during study 1, monopolar EMG signals were recorded with one electrode located on the *Gastrocnemius Medialis* (GM) and one electrode on the *Achilles* tendon. During study 2, bipolar EMG signals from the GM were recorded. All EMG signals were recorded using MP35 hardware (Biopac Systems Inc., Santa Barbara, CA) and dedicated software (BSL Pro Version 3.6.7, Biopac Systems Inc., Santa Barbara, CA). The myoelectric signal was amplified (gain = 1000 for bipolar and 200 for monopolar), filtered (30–1000 Hz) and recorded at a sampling frequency of 5000 Hz. Before electrode placement, the skin was lightly abraded and washed to remove surface layers of dead skin, hair, and oil; and a ground electrode was placed on the *patella*. The EMG activity was computed as the root mean square (RMS) of the signal. The RMS activity during the MVC was normalized by the amplitude of a concomitant superimposed M-wave (RMS/M).

#### Physiological monitoring

During all experiments, skin temperatures were monitored over the *Soleus* and GM muscles using a surface thermistor (Ellab, Hilleroed, Denmark) located aside the EMG electrodes. In addition, during study 1, rectal temperature was monitored using a rectal probe inserted 15 cm beyond the anal sphincter (MRB rectal probe, Ellab, Hilleroed, Denmark).

### Statistical analysis

Data were coded and analysed in PASW software v.18.0 (SPSS, Chicago, IL, US). The effect of heat exposure was analysed via repeated-measure ANOVA. ANOVA assumptions were verified preceding all statistical analyses; logarithmic transformations and Greenhouse-Geisser corrections were applied where appropriate. The level of statistical significance was set at p ≤ 0.05. Pearson’s product–moment correlation analysis was also used to assess the relationship between changes in torque (when appropriate) and neurophysiological measures.

Data are presented as mean ± SD along with the mean differences [95% confidence interval]. Effect-sizes are described in terms of partial eta-squared (η2; with η2 ≥ 0.06 representing a moderate difference and η2 ≥ 0.14 a large difference).

## Results

### Physiological response

During study 1, the core temperature was maintained at 39.0 ±0.2°C during the HOT-core trial by adjusting the room temperature as necessary in the range of 46-50 °C with a humidity of 35%. Consequently, core (+1.7 [1.5;1.9]°C, η2 = 0.96, p < 0.001) as well as skin (+10.3 [9.8;10.9]°C, η2 = 0.99, p < 0.001) temperatures were significantly higher in HOT-core than CON (Table 1). During study 2, skin temperatures were significantly higher in HOT-skin than CON (+10.7 [9.3;12.3]°C, η2 = 0.96, p < 0.001).

### Maximum voluntary contraction and voluntary activation

**Figure 1:**
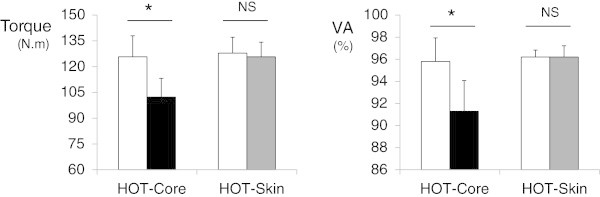
**Maximal voluntary contraction (MVC) in hot (dark columns) and control (white columns) conditions.** Left panel: Maximal torque produced during the MVCs. Right panel: Percentage of voluntary activation (VA) during the MVCs. HOT-core: passive hyperthermia in hot ambient conditions. HOT-skin: local modification of skin temperature. NS: No significant difference (η2 ≤ 0.08, p ≥ 0.36). * large (η2 = 0.67) and significant (p < 0.001) difference.

**Figure 2:**
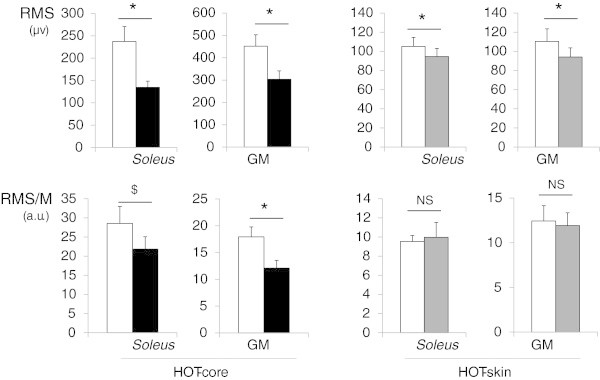
**Electromyographic activity in hot (dark columns) and control (white columns) conditions.** Left panel, HOT-core: passive hyperthermia in hot ambient conditions. Right panel, HOT-skin: local modification of skin temperature. RMS: Root Mean Square of the EMG signal. RMS/M RMS normalized by the amplitude of a M-wave. All data recorded during maximal voluntary contractions. NS: No significant differences (η2 = 0.01, p ≥ 0.77). $ and* large differences (η2 ≥ 0.18) with $ p = 0.15 and * p < 0.05.

Maximum voluntary torque was significantly reduced in HOT-core (−23 [−34;-13]N.m, η2 = 0.67, p < 0.001) but not HOT-skin (−2 [−7;+3]N.m, η2 = 0.08, p = 0.36) (Figure 1). VA was also reduced in HOT-core (−4.5 [−7.8;-1.2]N.m, η2 = 0.67, p < 0.001) but not HOT-skin (−0.0 [−1.9;+1.9]N.m, η2 = 0.00, p = 0.97) (Figure 1). RMS activity was lower in both HOT-core and HOT-skin, compared with respective control conditions (η2 > 0.44, p < 0.05 for both *Soleus* and GM, Figure 2). Once normalized to the amplitude of the concomitant M-wave, RMS/M remained lower (GM, -5.8 [−8.2;-3.4], η2 = 0.70, p < 0.001) or tended to be lower (*Soleus*, -6.7 [−16.1;+2.7], η2 = 0.18, p = 0.15) in HOT-core than CON. However, the RMS/M ratio were not different in HOT-skin as compared to CON (GM, -0.5 [−4.5;+3.5], η2 = 0.01, p = 0.78; *Soleus*, 0.4 [−2.7;+3.6], η2 = 0.01, p = 0.77) (Figure 2).

The changes in maximal torque observed in study 1 were correlated to the changes in VA (*r* = 0.61), but not with changes in EMG activity of the Soleus (*r* = −0.03) or of the GM (*r* = 0.34). In addition, the changes in both maximal torque and VA (*r* ≤ −0.6) but not the changes in RMS activity (−0.39 ≤ *r* ≤ 0.26) correlated to the changes in skin temperature.

### Torque / EMG relationship

**Figure 3:**
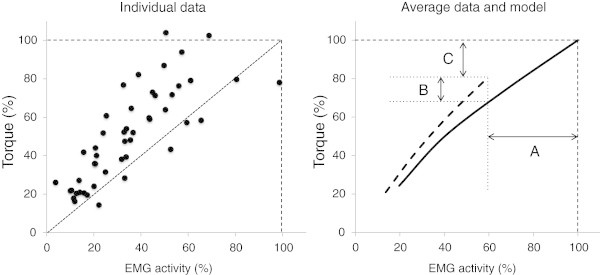
**Relation between plantar flexion torque and*soleus*EMG activity expressed in percentage of the maximal values in temperate environment.** Left panel: individual values during contraction ranging from 25% to 100% of MVC in a hot environment (HOT-core). Note that most values are above identity line (i.e., higher torque for a given EMG or lower EMG for a given torque in a hot environment as compare to a temperate environment). Right panel: Average relationship between torque and EMG in temperate (plain line) and hot (dashed line) environment. Note that the decrease in torque (C) is function of the decrease in EMG activity (A) minus the improvement in contractile properties (B).

When participants were required to produce a range of submaximal and maximal contractions during study 1, the EMG activity for both *soleus* and GM was linearly correlated to the torque for each individual (all 0.97 ≤ *r* ≤ 1.00). The slope of this relationship was significantly greater in HOT-core than CON for both the *soleus* (192 [36;347]Nm.mV^-1^, η2 = 0.40, p < 0.05) and the GM (55 [8;102]Nm.mV^-1^, η2 = 0.35, p < 0.05) (Table 1). Hence, a higher torque was produced in HOT-core than in CON for a given EMG activity (Figure 3).

The Torque / RMS ratio calculated during the MVC was significantly greater in HOT-core than CON for both the *Soleus* (166 [79;253]N.m.mV^-1^, η2 = 0.62, p < 0.001) and GM (53 [21;86]N.m.mV^-1^, η2 = 0.51, p = 0.004) (Table 1). This ratio was also greater in HOT-skin than CON for both the *Soleus* (119 [26;213]N.m.mV^-1^, η2 = 0.45, p = 0.02) and GM (147 [55;238]N.m.mV^-1^, η2 = 0.56, p = 0.005) (Table 1).

## Discussion

The current investigation aimed to determine the effect of environmental temperature upon muscle electrical activity during maximal and submaximal contractions. The data showed that passive hyperthermia reduced EMG activity as well as torque and voluntary activation during maximal isometric contractions. In addition, whereas increasing skin temperature did not affect force and voluntary activation, it decreased surface EMG activity.

The decrement in maximum voluntary torque with passive hyperthermia (study 1) was associated with a decrement in voluntary activation (Figure 1). The level of hyperthermia imposed in the study 1 (i.e., core temperature clamped at 39°C) was selected to induce significant neural alterations (Racinais and Cresswell 2013). Consequently, the current observations confirm previous reports that passive hyperthermia reduces voluntary drive to human skeletal muscles (Morrison et al. 2004; Thomas et al. 2006; Racinais et al. 2008; Périard et al. 2011). However, the current data demonstrated that warming the skin only (study 2) did not affect torque production or voluntary activation (Figure 1). These results confirm a previous study concluding that core temperature, rather than local thermal afferent input from the skin, mediates the alteration in neural drive (Thomas et al. 2006).

The novel finding of the current investigation is that RMS activity was decreased in both HOT-core and HOT-skin (Figure 2). This decrease was confirmed in two different muscles (*Soleus* and GM), using two different techniques (bipolar and monopolar EMG). Current literature suggests that this decrease might be partly related to changes in the spinal and peripheral transmission of the neural drive (Racinais et al. 2008 Racinais and Cresswell 2013). Indeed, a negative linear correlation has been documented between skin temperature and the amplitude, duration, area and latency of a compound action potential (Bolton et al. 1981). This observation suggests a shortening of the time that the voltage-gated sodium channels remain open with increasing temperature, leading to a decrease in the amplitude, duration and area of the action potential (Rutkove et al. 1997). Another possible mechanism would be a failure of the synaptic transmission at high temperatures as suggested by recent *in vitro* studies (Karunanithi et al. 1999 Kelty et al. 2002). However, the EMG responses followed a different pattern of response than torque and voluntary activation, suggesting that the decrement in recorded EMG might be affected by other mechanisms than a decrease in neural drive. In addition, the current data demonstrated that the changes in torque observed during study 1 correlated with changes in VA, but not with changes in EMG, suggesting that EMG is a poor predictor of the changes in muscle force production when environmental conditions change.

The different response in torque and EMG data in response to heat exposure might partly be due to a change in muscle contractile properties. *In vitro* studies have reported that maximum tetanic force can be improved by increasing temperature (Close and Hoh, 1968; Segal et al. 1986; Stephenson and Williams 1981), possibly by improving contractile protein binding (Stephenson and Williams 1981). Yet this may not be the case in all muscles groups (Segal et al. 1986),and increases in temperature across the standard range experienced *in vivo* (i.e., from 37 to 43°C) appears to not alter the absolute force of the muscle fibre (Place et al. 2009). Therefore, the current changes in torque / RMS relationship (study 1) and decrement in RMS when increasing skin temperature (study 2) might be related to methodological factors affecting the amplitude of the recorded RMS.

Previous studies have reported variable responses of EMG signal amplitude to temperature, with either an attenuation of EMG with cold-water immersion (Petrofsky and Lind 1980) or an increase of EMG amplitude in cold-air exposure (Winkel and Jotgenson 1991). In warm environments, the current data confirm that EMG activity can be reduced at a given force when temperature increases (Bell 1993; Racinais et al. 2005) (Figure 3). In addition, changing the temperature of the skin – electrode pairing also changes EMG activity recorded by surface electrodes (Figure 2). Importantly, such an effect was canceled by normalizing RMS activity to an electrically evoked M-wave (Figure 2).

Whereas RMS/M ratio were similar in HOT-skin with respect to CON (η2 = 0.01), they were largely (η2 ≥ 0.18) lowered in HOT-core as compared to a temperate CON. Thus, the normalization of EMG signals by the amplitude of an electrically evoked action potential appears to be a suitable way to circumvent the effect of a modification in skin – electrode temperature, without blunting the central effects of hyperthermia. However, the M-wave is also dependent on the transmission of the signal through the sarcolemma and the neuromuscular junction, and such normalization might blunt the physiological effects of hyperthermia on the peripheral nervous system (Racinais et al. 2008) and should be interpreted with caution.

A peripheral vasodilatation shifting more fluid between the EMG signal and the surface (Bell 1993), and an increase in signal conduction velocity (Racinais et al. 2008) might also have affected the EMG recording in a hot environment. However, these mechanisms do not systematically affect the EMG data as a recent study reported that EMG activity during both the relaxation and the motor phase at a given power output were not affected by hot ambient conditions (Racinais and Girard 2012). Therefore, it is recommended that EMG recordings should be obtained from other muscles, or from resting and submaximal conditions to ascertain the stability of the recording condition, before conclusions on the effect of hot ambient conditions on maximal neural drive and force capacity may be drawn.

Finally, the current data demonstrated that passive hyperthermia modified the slope of the torque / EMG relationship (Figure 3). This modification corresponded to a higher torque produced in hot rather than temperate conditions for a given RMS activity (Figure 3). These changes are supported by a higher torque / RMS ratio during the MVCs in both HOT-core and HOT-skin, as compared to control conditions (Table 1). Following the model displayed in Figure 3, the decrement in force during passive hyperthermia (arrow C) represents the resultant of a decrease in muscle activity (arrow A), which is partly compensated for by an improvement in neuromuscular efficiency (arrow B) (Figure 3). Consequently, it is not possible to infer the force produced by a muscle in hot environments based on a comparison of its EMG activity with reference values obtained in a temperate environment. As displayed in Table 1, the slope of the torque / RMS relationship suggests that participants were able to produce 576 (±283) N.m of torque per mV of electrical activity of the *soleus* in a temperate environment whereas this value increased by 33% to 768 (±204) N.m in hot ambient conditions.

## Conclusion

In summary, the present investigations demonstrate that passive hyperthermia in hot ambient conditions shifts the torque / EMG relationship to the left. Consequently, EMG inferred data in hot environments are likely to overestimate the decrement in performance.
